# Pollution load index for heavy metals in Mian-Ab plain soil, Khuzestan, Iran

**DOI:** 10.1016/j.dib.2017.10.017

**Published:** 2017-10-12

**Authors:** Sahand Jorfi, Rohangiz Maleki, Neemat Jaafarzadeh, Mehdi Ahmadi

**Affiliations:** a Environmental Technologies Research Center, Ahvaz Jundishapur University of Medical Sciences, Ahvaz, Iran; b Department of Environmental Health Engineering, Ahvaz Jundishapur University of Medical Sciences, Ahvaz, Iran

**Keywords:** Soil pollution, Pollution load index, Heavy metals, Mian-Ab plain

## Abstract

Soil pollution by heavy metals is a major concern in agricultural area. Potential impact of heavy metals in agricultural soil on human health by accumulating in food chain demonstrated elsewhere.

In this regard Mian-Ab plain as a major agricultural site of Khuzestan province considered for Arsenic, cadmium and lead concentration as the main potential toxic pollutants in soil. 50 topsoil samples were collected and analyzed by inductively coupled plasma mass spectrometry (ICP-MS). Also Contamination level of selected heavy metals in Mian-Ab Plain, was assessed by single factor contaminant index (PI) and pollution load index (PLI). Results show mean concentration of arsenic, cadmium and lead were 2.52, 0.30 and 7.21 mg kg^−1^. Base on PLI results 12 point (24%) of the studied area show moderately polluted and 38 point (76%) show unpolluted area.

**Specifications Table**

**Table:** 

Subject area	*Environmental monitoring*
More specific subject area	*Soil pollution monitoring*
Type of data	*Table and Figures*
Data format	*Raw and Analyzed*
Experimental factors	*50 soil samples analyzed for cadmium, Arsenic and Lead concentration*
Experimental features	*Upon soil sampling from selected point, cadmium, Arsenic and Lead concentration in soil samples measured by inductivity coupled plasma, descriptive statistics' was done and distribution maps of selected heavy metals prepared. Also pollution level was assessed using single pollution indices and pollution load index.*
Data source location	*Shushtar, Khuzestan, Iran, 31°40′ and* 32°05′ *N* and 48°45′ and 49°00′ *E*
Data accessibility	*Data are available in the article*

**Value of the data**

• Arsenic, cadmium and lead are the main potentially toxic pollutants.
• Mian-Ab Plain is one of the most agricultural sites in Khuzestan province.
• Soil pollution by heavy metals is the major concern in developing countries.

## 1 Data

**Fig. 1:**
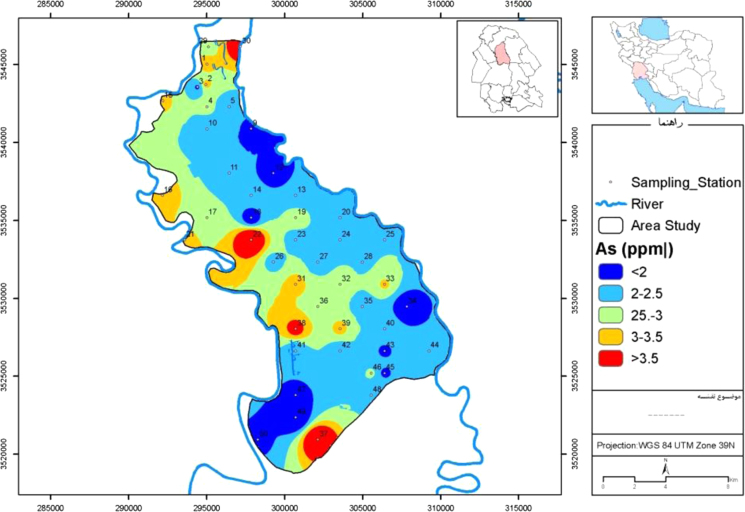
Map of arsenic distribution in Mian-Ab plain.

**Fig. 2:**
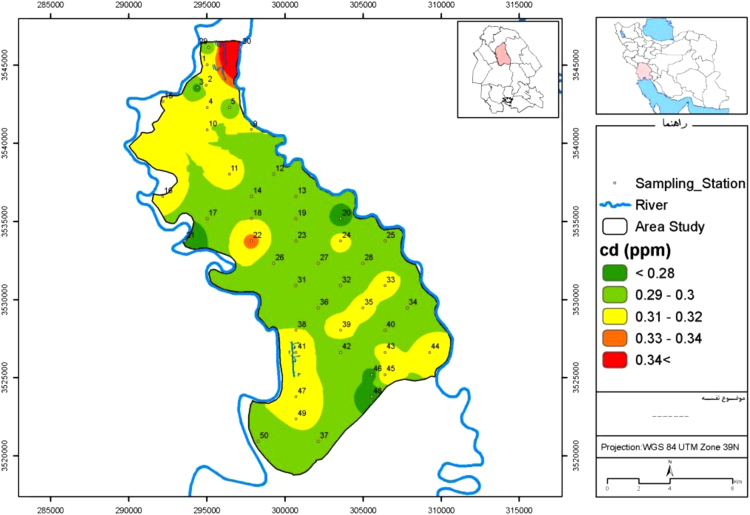
Map of cadmium distribution in Mian-Ab plain.

**Fig. 3:**
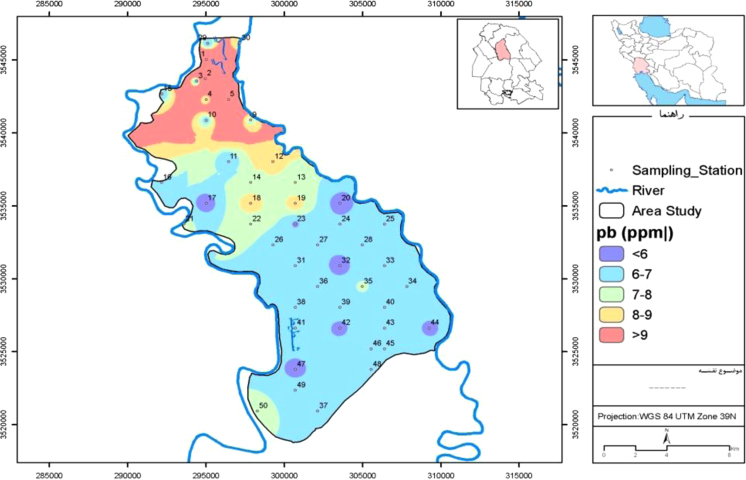
Map of lead distribution in Mian-Ab plain.

Table 1 shows Descriptive statistics of selected heavy metals in Mian-Ab plain soil also Fig. 1, Fig. 2, Fig. 3 show distribution of Arsenic, cadmium and lead in Mian-Ab plain respectively. Table 2 shows single pollution indices and pollution load index of selected heavy metals.

**Table 1:** Descriptive statistics of selected heavy metals in Mian-Ab plain soil.

**Item**		**Ar (mg kg^−1^)**	**Cd (mg kg^−1^)**	**Pb (mg kg^−1^)**
*N*		50	50	50
Mean		2.5220	0.3030	7.2140
Std. Error of Mean		0.11785	0.00758	0.45062
Median		2.3500	0.3000	6.6500
Mode		2.10	0.31	6.00
Std. Deviation		0.83331	0.05361	3.18639
Variance		0.694	0.003	10.153
Skewness		1.272	5.712	5.337
Std. Error of Skewness		0.337	0.337	0.337
Kurtosis		2.397	37.353	31.827
Std. Error of Kurtosis		0.662	0.662	0.662
Range		4.30	0.40	21.60
Minimum		1.00	0.25	5.40
Maximum		5.30	0.65	27.00
Sum		126.10	15.15	360.70
Percentiles	25	2.0000	0.2800	6.0000
	50	2.3500	0.3000	6.6500
	75	2.8750	0.3100	7.2000

**Table 2:** Single pollution indices (PI) and pollution load index (PLI) for selected heavy metals in Mian-Ab plain.

**Sample ID**	***X***	***Y***	***Z***	**AS**		**Cd**		**Pb**		**PLI**
				**Conc.**	**PI**	**Conc.**	**PI**	**Conc.**	**PI**	
1	295,018	3545,035	37	3.2	1.269	0.31	1.02	14.7	2.0	1.4
2	294,968	3543,723	33	3.2	1.280	0.31	1.03	27.0	3.8	1.7
3	294,425	3543,546	45	1.9	0.760	0.27	0.90	5.4	0.8	0.8
4	295,018	3542,297	35	2.7	1.080	0.31	1.03	7.7	1.1	1.1
5	296,443	3542,297	52	2.0	0.800	0.29	0.97	7.2	1.0	0.9
6	293,595	3540,873	53	2.1	0.840	0.27	0.90	5.6	0.8	0.8
7	290,747	3539,449	54	2.4	0.960	0.32	1.07	5.9	0.8	0.9
8	295,019	3539,449	49	2.1	0.840	0.35	1.17	6.0	0.8	0.9
9	297,867	3540,873	53	1.8	0.720	0.30	1.00	7.4	1.0	0.9
10	295,018	3540,870	38	2.0	0.800	0.30	1.00	6.5	0.9	0.9
11	296,443	3538,025	52	2.2	0.880	0.31	1.03	6.0	0.8	0.9
12	299,291	3538,025	52	1.5	0.600	0.28	0.93	8.9	1.2	0.9
13	300,715	3536,601	52	2.1	0.840	0.28	0.93	7.0	1.0	0.9
14	297,867	3536,601	52	2.4	0.960	0.29	0.97	7.6	1.1	1.0
15	292,197	3542,670	35	3.2	1.280	0.31	1.03	6.4	0.9	1.1
16	292,171	3536,601	54	3.4	1.360	0.32	1.07	6.7	0.9	1.1
17	295,019	3535,177	40	2.6	1.040	0.28	0.93	5.5	0.8	0.9
18	297,867	3535,177	55	1.4	0.560	0.30	1.00	8.7	1.2	0.9
19	300,715	3535,177	55	2.8	1.120	0.30	1.00	8.7	1.2	1.1
20	303,563	3535,177	44	2.3	0.920	0.27	0.90	5.6	0.8	0.9
21	293,595	3533,753	25	3.2	1.280	0.25	0.83	7.2	1.0	1.0
22	297,867	3533,753	25	5.3	2.120	0.33	1.10	7.6	1.1	1.4
23	300,715	3533,753	43	2.3	0.920	0.28	0.93	5.9	0.8	0.9
24	303,563	3533,753	43	2.2	0.880	0.31	1.03	6.0	0.8	0.9
25	306,411	3533,753	43	2.5	1.000	0.29	0.97	6.6	0.9	1.0
26	299,291	3532,329	43	2.2	0.880	0.29	0.97	6.3	0.9	0.9
27	302,139	3532,329	43	2.1	0.840	0.30	1.00	6.3	0.9	0.9
28	304,987	3532,329	42	2.4	0.960	0.29	0.97	7.0	1.0	1.0
29	297,185	3546,158	42	2.8	1.120	0.28	0.93	6.1	0.8	1.0
30	297,185	3546,158	42	4.4	1.760	0.65	2.17	7.9	1.1	1.6
31	300,715	3530,905	42	3.3	1.320	0.28	0.93	6.8	0.9	1.1
32	303,563	3530,905	45	2.8	1.120	0.28	0.93	5.5	0.8	0.9
33	306,411	3530,905	46	3.1	1.240	0.31	1.03	6.1	0.8	1.0
34	307,835	3529,481	46	1.3	0.520	0.28	0.93	7.0	1.0	0.8
35	304,987	3529,481	45	2.2	0.880	0.31	1.03	7.2	1.0	1.0
36	302,139	3529,481	45	2.7	1.080	0.30	1.00	6.7	0.9	1.0
37	302,139	3520,937	42	4.8	1.920	0.30	1.00	7.0	1.0	1.2
38	300,715	3528,057	45	3.9	1.560	0.30	1.00	6.7	0.9	1.1
39	303,563	3528,057	45	3.2	1.280	0.31	1.03	6.4	0.9	1.1
40	306,411	3528,057	44	2.4	0.960	0.28	0.93	6.0	0.8	0.9
41	300,715	3526,633	43	2.0	0.800	0.31	1.03	6.0	0.8	0.9
42	303,563	3526,633	44	2.1	0.840	0.28	0.93	5.8	0.8	0.9
43	306,411	3526,633	44	1.9	0.760	0.31	1.03	6.7	0.9	0.9
44	309,259	3526,633	45	2.2	0.880	0.31	1.03	5.9	0.8	0.9
45	306,411	3525,209	45	1.9	0.760	0.32	1.07	6.7	0.9	0.9
46	305,563	3525,209	45	2.6	1.040	0.27	0.90	6.9	1.0	1.0
47	300,715	3523,785	45	1.7	0.680	0.32	1.07	5.8	0.8	0.8
48	305,563	3523,785	44	2.5	1.000	0.26	0.87	6.1	0.8	0.9
49	300,715	3522,361	43	1.0	0.400	0.30	1.00	6.2	0.9	0.7
50	298,291	3520,937	43	1.8	0.720	0.28	0.93	7.8	1.1	0.9

## 2 Experimental design, materials and methods

### 2.1 Sampling and analysis procedure

**Fig. 4:**
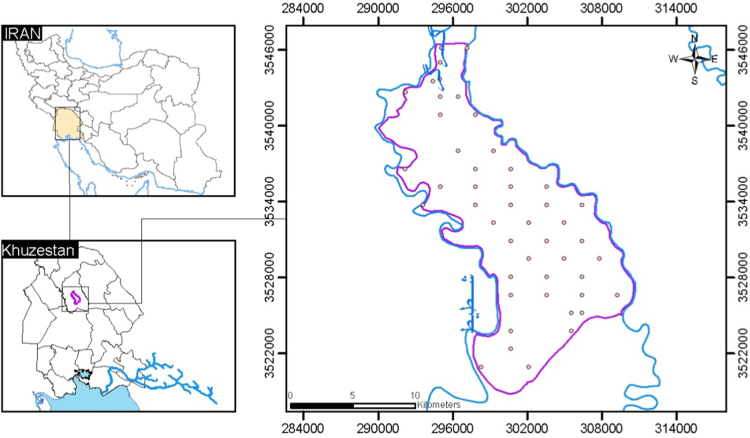
Geological map of Mian-Ab plain and sampling points in the study area.

Mian-Ab plain has an area of 28,100 ha, and is located between 31°40′ and 32°05′ northern latitudes and 48°45′ and 49°00′ eastern longitudes, north of Khuzestan province, and south west of Iran. Mian-Ab Plain is bounded between Shotait River and Gargar River [1]. Geologically, the study area is widely characterized by quaternary unconsolidated alluvial sediment. A systematic sampling procedure was performed to provide a sampling scheme over the entire plain. Therefore, in March 2016 (dry season), 50 topsoil samples (0–20 cm) were collected using a stainless steel hand auger (Fig. 4). In each sampling point, a total of 1 ± 0.5 kg of soil was taken from the mixed samples using a quartile method. The collected soil samples were stored in polyethylene bags until transport to the laboratory.

In the laboratory, all samples were air-dried at room temperature and sieved through a 2-mm sieve. Finally, the dried soil samples were sent to the MS Analytical, Canada. The soil samples were digested using the Aqua Regia, and the concentration of 3 potentially toxic elements (PTEs) including (As, Cd, Pb) was measured using inductively coupled plasma mass spectrometry (ICP-MS) [2], [3], [4], [5], [6].

### 2.2 Quality assurance (QA) and quality control (QC)

Analytical duplicates/replicates, standard reference material (OREAS 24b, and GBM908-10), and blank reagents were used for QA/QC. Recoveries ranged from 95.6% to 98.74% and the detection limits for As, Cd and Pb were 0.1, 1.88 and 0.2 respectively.

### 2.3 Soil pollution assessment

Contamination level of selected heavy metals in Mian-Ab Plain, was assessed by single factor contaminant index (PI) and pollution load index (PLI) using Eqs. (1), (2) [7].

(1) $$\mathrm{PI}=C_{n}/B_{n}$$

(2) $$\mathrm{PLI}=\sqrt{\mathrm{PI}1\times \mathrm{PI}2\ldots .\mathrm{PIn}}$$

Where PI is the single factor pollution index of each metal, C_n_ and B_n_ is the concentration of metal in the soil sample and background, respectively (mg/Kg).

PI < 1 (Non polluted); 1 ≤ PI < 2 (Slight polluted); 2 ≤ PI < 3 (Moderately polluted); PI < 3 (Highly polluted).

PLI is pollution load index, n is the number of pollutant assesses (i.r., 3). PI is the single factor pollution index of each metal.

PLI = 0 (background concentration); 0 < PLI ≤ 1 (Unpolluted); 1 < PLI ≤ 2 (Moderately to unpolluted); 2 < PI ≤ 3 (Moderately polluted); 3 < PI ≤ 4 (Moderately to highly polluted); 4 < PI ≤ 5 (Highly polluted); PI > 3 (Very highly polluted).

## References

[1] Shahsavari A.A., Khodaei K., Asadian F., Ahmadi F., Zamanzadeh S.M. (2012). Groundwater pesticides residue in the southwest of Iran-Shushtar plain. Environ. Earth Sci..

[2] Jaafarzadeh N., Amiri H., Ahmadi M. (2012). Factorial experimental design application in modification of volcanic ash as a natural adsorbent with Fenton process for arsenic removal. Environ. Technol..

[3] Jaafarzadeh N., Ahmadi M., Amiri H., Yassin M.H., Martinez S.S. (2012). Predicting Fenton modification of solid waste vegetable oil industry for arsenic removal using artificial neural networks. J. Taiwan Inst. Chem. Eng..

[4] Ahmadi M., Teymouri P., Setodeh A., Mortazavi M.S., Asgari A. (2011). Adsorption of Pb (II) from aqueous solution onto lewatit FO36 nano resin: equilibrium and kinetic studies. Environ. Eng. Manag. J..

[5] Teymouri P P., Jaafarzadeh N., Mostoufi A., Amiri H., Alavi N., Dinarvand M., Ahmadi M. (2017). Effect of pretreatment on Ceratophyllum demersum for enhanced biosorption of Cr (VI) and Cd (II). Environ. Eng. Manag. J..

[6] Ahmadi M M., Kouhgardi E., Ramavandi B. (2016). Physico-chemical study of dew melon peel biochar for chromium attenuation from simulated and actual wastewaters. Korean J. Chem. Eng..

[7] Ahmadi M., Jorfi S., Azarmansuri A A., Jaafarzadeh N., Mahvi A.H., Darvishi Cheshmeh Soltani R., Akbari H., Akhbarizadeh R. (2017). Zoning of heavy metal concentrations including Cd, Pb and As in agricultural soils of Aghili plain, Khuzestan province, Iran. Data Brief.

