# Supplementary material for: Pollution load index for heavy metals in Mian-Ab plain soil, Khuzestan, Iran
Source: Data Brief. 2017 Oct 12;15:584–90. doi: 10.1016/j.dib.2017.10.017 (PMC5653243; doi:10.1016/j.dib.2017.10.017)
Supplement: Supplementary file 1 — Transparency document [file mmc1.docx]

**Conflict of interest**

No conflict of interest associated with this data article.

**Acknowledgements**

This paper is issued from thesis of Rohangiz Maleki and financial support was provided by Ahvaz Jundishapur University of Medical Sciences (Grant no: ETRC9445).
